# Combining high-throughput micro-CT-RGB phenotyping and genome-wide association study to dissect the genetic architecture of tiller growth in rice

**DOI:** 10.1093/jxb/ery373

**Published:** 2018-10-30

**Authors:** Di Wu, Zilong Guo, Junli Ye, Hui Feng, Jianxiao Liu, Guoxing Chen, Jingshan Zheng, Dongmei Yan, Xiaoquan Yang, Xiong Xiong, Qian Liu, Zhiyou Niu, Alan P Gay, John H Doonan, Lizhong Xiong, Wanneng Yang

**Affiliations:** 1 National Key Laboratory of Crop Genetic Improvement and National Center of Plant Gene Research, Agricultural Bioinformatics Key Laboratory of Hubei Province, College of Informatics, and College of Engineering, Huazhong Agricultural University, Wuhan, China; 2 Britton Chance Center for Biomedical Photonics, Wuhan National Laboratory for Optoelectronics, and Key Laboratory of Ministry of Education for Biomedical Photonics, Department of Biomedical Engineering, Huazhong University of Science and Technology, Wuhan, China; 3 The National Plant Phenomics Centre, Institute of Biological, Environmental and Rural Sciences, Aberystwyth University, Aberystwyth, UK

**Keywords:** Dynamic phenotyping, GWAS, high throughput, longitudinal traits, micro-CT-RGB, plant phenomics, rice tiller, tiller traits

## Abstract

Manual phenotyping of rice tillers is time consuming and labor intensive, and lags behind the rapid development of rice functional genomics. Thus, automated, non-destructive methods of phenotyping rice tiller traits at a high spatial resolution and high throughput for large-scale assessment of rice accessions are urgently needed. In this study, we developed a high-throughput micro-CT-RGB imaging system to non-destructively extract 739 traits from 234 rice accessions at nine time points. We could explain 30% of the grain yield variance from two tiller traits assessed in the early growth stages. A total of 402 significantly associated loci were identified by genome-wide association study, and dynamic and static genetic components were found across the nine time points. A major locus associated with tiller angle was detected at time point 9, which contained a major gene, *TAC1*. Significant variants associated with tiller angle were enriched in the 3ʹ-untranslated region of *TAC1.* Three haplotypes for the gene were found, and rice accessions containing haplotype H3 displayed much smaller tiller angles. Further, we found two loci containing associations with both vigor-related traits identified by high-throughput micro-CT-RGB imaging and yield. The superior alleles would be beneficial for breeding for high yield and dense planting.

## Teaser

Combining high-throughput micro-CT-RGB phenotyping with genome-wide association study reveals the genetic architecture of tiller development in the *indica* subpopulation of rice.

## Introduction

Rice is one of the most important food crops both in China and worldwide (Zhang, 2008). Selecting plants with the ideal tiller structure, particularly in terms of tiller angle and number. was a key issue in domesticating rice and improving its yield (Wang *et al.*, 2008). With the rapid development of functional genomics and molecular breeding, the ability to quickly screen thousands of lines for targeted phenotypic traits under different growth conditions is important (Fiorani and Schurr, 2013). Manual phenotyping methods of tiller traits, such as counting tillers, determining whether they are potentially reproductive, and measuring the tiller angles with a protractor, are time consuming and labor intensive, and potentially damaging, and so delay the development of rice genomics for these important characters (Houle *et al.*, 2010; Furbank *et al.*, 2011). To bridge this gap, progress in the development of high-throughput phenotyping technology is required to accelerate gene discovery and assist with rice breeding (Yang *et al.*, 2013).

Tiller numbers and angles are two key components of plant architecture that affect the grain yield of rice (Springer, 2010). Tiller number largely determines the panicle number, a key component of yield. Many tiller-related genes have been identified in recent years, such as *MOC1*(Li *et al.*, 2003), *OsTB1*(Takeda *et al.*, 2003), and *IPA1* (Jiao *et al.*, 2010). These genes are involved in the initiation and outgrowth of axillary meristems, and in the auxin and strigolactone signaling pathway that controls rice tillering (Li *et al.*, 2003; Takeda *et al.*, 2003; Guo *et al.*, 2013). MicroRNAs are also involved in rice tillering by regulating the expression of target genes (Xia *et al.*, 2012; Liang *et al.*, 2014). *MOC1*, which was first isolated and characterized in the context of the control of rice tillering, positively regulates tiller number by initiating axillary buds and promoting their outgrowth (Li *et al.*, 2003). Tiller angle, which determines the ideal plant architecture and thus grain yield (Sang *et al.*, 2014), has undergone improvement during domestication. Small tiller angles make plants more efficient in photosynthesis and increase radiation use efficiency, and also allow denser planting during rice cultivation (Yu *et al.*, 2007). Several tiller-angle related genes, such as *TAC1*, *TAC3*, *OsLIC*, and *PROG1*, have been identified and characterized (Yu *et al.*, 2007; Jin *et al.*, 2008; Wang *et al.*, 2008; Dong *et al.*, 2016). *TAC1* is a major gene that was identified by forward genetics as positively controlling tiller angle (Yu *et al.*, 2007). A variant in the 3ʹ-untranslated region (UTR) of the gene changes the mRNA level, and higher mRNA levels contribute to a larger tiller angle. Based on previous studies, nucleotide diversity in *TAC1* is low, and only one single nucleotide polymorphism (SNP) in the coding region has been found, resulting in synonymous substitution in 113 cultivated rice varieties. The small-angle allele of *TAC1* exists only in the *japonica* accessions (Jiang *et al.*, 2012).

Recent improvements in detector arrays and micro-focus X-ray tubes have allowed the development of computed tomography (CT) to produce micro-CT (also termed high-resolution CT), which can non-invasively scan the internal three-dimensional (3D) structure of plant tissues and organs (Stuppy *et al.*, 2003). With the addition of appropriate methods and specific image analysis, both 3D visualization and volumetric measurements of plant tissues or organs can be obtained. High-resolution CT was able to visualize the 3D vessel networks of dry grapevine stems (Brodersen *et al.*, 2011) and vascular bundles of cut maize stalks (Du *et al.*, 2016), in which the reconstructed results were close to those acquired by electron microscopy. Micro-CT can also be used to quantify floral traits (numbers of pollen grains and ovules; Staedler *et al.*, 2018) and wheat grain traits (Hughes *et al.*, 2017), and thus has the potential to replace labor-intensive manual counting. Using different sample holders and appropriate X-ray energies, 3D structures of different sizes, including plant buds and inflorescences, barley spikes, and whole barley plants, can be measured using micro-CT (Tracy *et al.*, 2017); this application demonstrated that micro-CT can be used to measure successive flower development and spike growth in a non-destructive manner. Moreover, the tiller number and spike length of barley can be manually counted using the projected X-ray images (Tracy *et al.*, 2017). However, manual counting using projected X-ray images is not suitable for tiller counting in large populations (particularly those with high tiller numbers), and tiller growth traits cannot be measured on projected X-ray images.

The rapid development of high-throughput phenotyping technologies such as field high-throughput phenotyping (Jose *et al.*, 2014) and cell to whole-plant phenotyping (Stijn *et al.*, 2013) has accelerated the genetic mapping of important agronomic traits in crops. Using the precision field phenotyping platforms, quantitative trait loci for controlling biomass were identified in triticale (Busemeyer *et al.*, 2013). The panicle-related image-analysis pipeline PANorama promoted the genetic dissection of rice panicle traits (Crowell *et al.*, 2014). Given the abundant genetic variation in natural populations, combinations of high-throughput phenotyping and genome-wide association studies (GWAS) have been conducted to reveal the natural genetic variation and to dissect the genetic architecture of complex traits such as biomass, grain yield, leaf traits, panicle, and salinity tolerance (Yang *et al.*, 2014, 2015; Al-Tamimi *et al.*, 2016; Crowell *et al.*, 2016).

In the present work, we developed a high-throughput micro-CT-RGB (HCR) imaging system combining CT and RGB imaging in one chamber to extract tillering traits, with high spatial resolution (97 μm) and high efficiency (~310 plants per day). A panel containing 234 rice accessions was phenotyped non-destructively at nine time points during the tillering process, and 739 traits were extracted by HCR and used to perform GWAS. In addition, we analyzed the relationship between tiller senescence and drought resistance, and dynamically screened changes in tillering under drought stress. Our results demonstrate that combining HCR and GWAS provides new insight into the genetic basis of rice tillering and architecture.

## Materials and methods

### Plant material and experimental design

In this study *indica* rice accessions were used because of their higher genetic diversity than *japonica* subpopulations (Huang *et al.*, 2010). The experiment aimed to measure as many genotypes as possible. Measurements of replicate plants for each genotype had high repeatability (*w*^2^>0.89) based on data from our previous study (Guo *et al.*, 2018) using the same rice accessions under similar cultural and environmental conditions to those of the current study. On this basis, in the current study one rice plant of each genotype was measured with the HCR imaging system. Genotype and phenotype information for the 234 accessions was retrieved from the RiceVarMap v2.0 website (http://ricevarmap.ncpgr.cn/v2/). Detailed cultivar information for the 234 accessions was obtained from the same website (http://ricevarmap.ncpgr.cn/v2/cultivars/). The genotype identity, cultivar name, and all phenotypic traits are tabulated in Dataset S1 available at Dryad Digital Repository (https://doi.org/10.5061/dryad.gm18v5f; Wu *et al.*, 2019). Seeds from the accessions were sown in the field on 25 May 2015 and transplanted into pots on 16 June 2015. Each pot was filled with 5 kg soil (pH 5.45, total nitrogen 0.241 g kg^–1^, total potassium 7.20 g kg^–1^, total phosphorus 0.74 g kg^–1^, alkali-hydrolyzable nitrogen 144.06 mg kg^–1^, available potassium 188.64 mg kg^–1^, available phosphorus 16.81 mg kg^–1^, organic matter 46.55 g kg^–1^). During the tiller elongation stage (~41–67 days after sowing), the 234 accessions were automatically measured every 3 days (nine times in total) using HCR. At the mature stage, the effective tiller number was counted manually from the reconstructed transverse section of tiller images. After harvest, 203 plants were oven-dried and weighed to obtain the shoot dry weight. Then, the 203 rice plants were threshed and then inspected by using a yield traits scorer (Yang *et al.*, 2014) to measure the grain yield. In addition, 35 plants and plastic pipes (see Fig. S1 available at Dryad) were processed by HCR and then manually measured to assess the comparability of HCR with manual measurements.

Comparisons of the manual and HCR measurements were made using mean absolute percentage error (MAPE) and root mean square error (RMSE), defined as follows:

$$MAPE=\frac{1}{n}\sum_{i=1}^{n}\frac{\left|x_{ai}-x_{mi}\right|}{x_{mi}}\times 100\%$$ (1)

$$RMSE=\sqrt{\frac{1}{n}\sum_{i=1}^{n}{\left(x_{ai}-x_{mi}\right)}^{2}}$$ (2)

Where *n* is the total number of measurements, the subscript a refers to HCR measurements, the subscript m refers to manual measurements, and the subscript i refers to the one of the *n* measurements on a nominated character.

To investigate the implications of tiller senescence for yield and drought resistance, three drought-resistance traits (stay-green trait, leaf-rolling trait, and leaf water content) of 100 *indica* rice accessions assessed in our previous work were re-analyzed (Guo *et al.*, 2018) and compared with the tiller senescence of the same 100 accessions in the present study. Tiller senescence was calculated as:

$$\text{Tiller senescence}=\frac{\text{TN\_9-TN\_Effective}}{\text{TN\_9}}$$ (3)

Where TN_9 is the tiller number at time point 9 (late tillering stage) and TN_Effective is the effective tiller number at the mature stage, which is distinguished by the presence of a pith-filled void that indicates that these tillers have booted.

In addition, to dynamically screen changes in the tillers under drought stress, one rice variety, Zhonghua 11 (*Oryza sativa* L. ssp. *japonica*), was inspected with the HCR system every day for 13 consecutive days, through progressive drought stress and rewatering.

The experimental design is shown in Fig. S2 available at Dryad. The environmental conditions of rice plant growth, including radiation, temperature, humidity, vapor pressure, wind speed, and total rainfall per day are shown in Fig. S3 available at Dryad.

### Main components and configuration of HCR

**Fig. 1.**
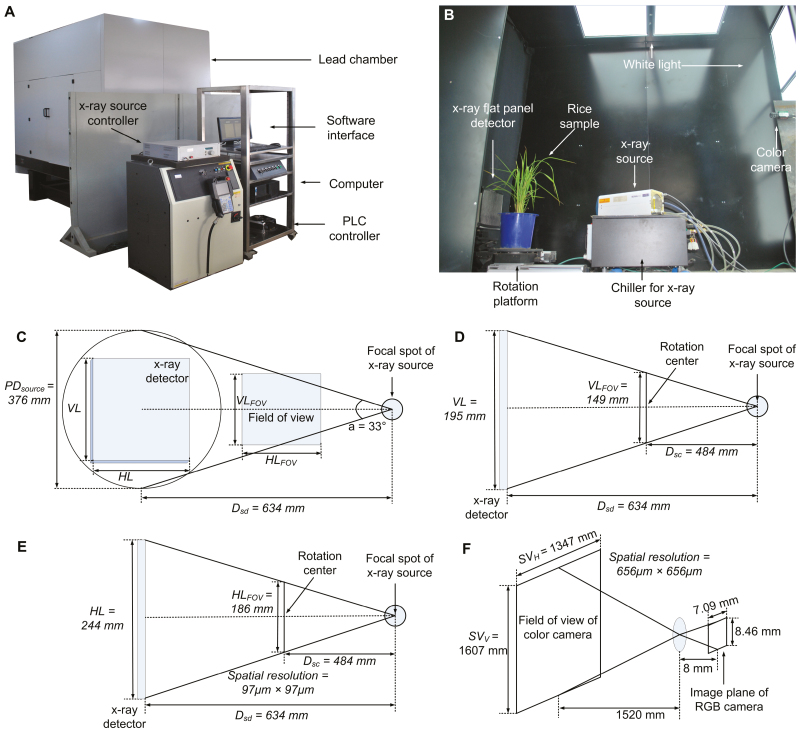
Main components and configuration of HCR. (A) Prototype of the HCR system. PLC, Programmable logic controller. (B) Layout of the inspection unit. (C) The cone angle of the focal spot of the X-ray source is 33º, and the distance between the X-ray source and the detector (*D*_sd_) is 634 mm, thus the projection diameter of the X-ray source (*PD*_source_) is 376 mm. (D) The distance between the focal spot of the X-ray source and the X-ray flat panel detector (*D*_sd_), the distance between the X-ray source and the rotation center (*D*_sc_), and the vertical length of the X-ray flat panel detector (*VL*) are 634 mm, 484 mm, and 195 mm, respectively, thus the vertical length of the field of view (*VL*_FOV_) is 149 mm. (E) The distance between focal spot of the X-ray source and the X-ray flat panel detector (*D*_sd_), the distance between the X-ray source and the rotation center (*D*_sc_), and the horizontal length of the X-ray flat panel detector (*HL*) are 634 mm, 484 mm, and 244 mm, respectively, thus the horizontal length of field of view (*HL*_FOV_) is 186 mm. The spatial resolution can be calculated as 97 μm×97 μm. (F) For the RGB camera, the object distance is 1520 mm, the image distance is 8 mm, and the image plane area is 7.09 mm×8.46 mm, thus the field of view is 1607 mm (vertical, *SV*_V_)×1347 mm (horizontal, *SV*_H_).

The bimodal imaging system, including micro-CT and RGB imaging, was developed to non-destructively measure 75 phenotypic traits. The HCR consists of nine main elements: an X-ray source (Nova600, Oxford Instruments, UK), an X-ray source chiller (Nova600, Oxford Instruments, UK), an X-ray flat panel detector (PaxScan 2520DX, Varian Medical Systems, Inc., USA), an RGB camera (AVT Stingray F-504B, Allied Vision Technologies Corporation, Germany), a white light, a rotation platform (MSMD022G1U, Panasonic, Japan), a lead chamber, a computer (M6600N, Lenovo, China), and a programmable logic controller (CP1H, OMRON Corporation, Japan) (see Fig. 1A, B). Diagrams showing the configuration of the HCR system are provided in Fig. 1C–F. The trade-off between the CT image resolution and CT scan area was set to provide an image of a whole plant in a single scan with spatial resolution of 97 μm and a field of view (FOV) 149 mm high×186 mm wide. The RGB imaging system’s FOV is 1607 mm high×1347 mm wide and its spatial resolution is 656 μm. The main specifications of the HCR inspection unit are shown in Table S1 available at Dryad.

### Image acquisition and analysis of HCR

**Fig. 2.**
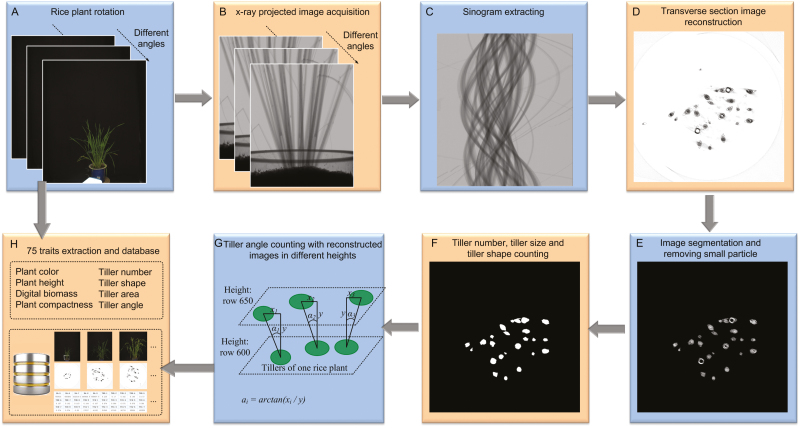
Image analysis pipeline of HCR. (A, B) As the rice sample rotated, 20 color images and 380 X-ray projected images at different angles were acquired synchronously; (C) one row of X-ray projected images at the same height as the 380 X-ray projected images, which formed a sinogram covering 380 orientations, was selected (step 0.6°, total angle 0.6°×380, ~228°); (D) a conventional filtered back-projection (FBP) algorithm was applied to obtain a reconstructed transverse section image of the rice tillers; (E, F) after image segmentation and the removal of small particles, the tiller number, size, and shape were counted; (G) two transverse tiller images were reconstructed at two different heights (row 600 and row 650), and the tiller angle was calculated using the spatial location of the central point of the rice tiller images; (H) 75 phenotypic traits (including plant color, plant height, digital biomass, plant compactness, tiller number, shape, area, and angle) were extracted and stored in a database, which also included RGB images and micro-CT images.

The image acquisition and analysis pipeline was developed using LabVIEW 8.6 (National Instruments, Inc., USA), and the control of the X-ray panel detector was developed with calling the dynamic link library provided by Varian Medical Systems, Inc., USA. The automated image-acquisition software controlled the collection of 380 X-ray projected images (step 0.6°, total angle 0.6°×380, ~228°, Fig. 2B) and 20 color images (step 11.4°, total angle 11.4°×380, ~228°; Fig. 2A) collected in parallel while the plant was in the imaging chamber. Tiller traits were acquired by the following steps (Fig. S4 available at Dryad): (i) one series of 380 X-ray projected images at the same height was selected to form a sinogram (Fig. 2C) covering 380 orientations (step 0.6°, total angle 0.6°×380, ~228°); (ii) a conventional filtered back-projection algorithm was applied to reconstruct the transverse section of the rice tiller (Fig. 2D); (iii) erosion and dilation were then used to remove the reconstruction artefacts and a particle filter with irregular shape was applied to remove the leaf blades in the reconstructed image. After image segmentation and small particle removal (Fig. 2E), the tiller number, size, and shape were automatically measured (Fig. 2F); (iv) then, two transverse tiller images were reconstructed at two different heights (row 600 and row 650, representing ~50–55 mm height from the soil surface), and the tiller angle (mean, maximum, and SD of the tiller angles in one rice plant) was calculated using the spatial location of the central point of the rice tiller images (Fig. 2G). Finally, 58 phenotypic traits, including plant color, plant height, digital biomass, and plant compactness, were obtained from the RGB images and analyses (Yang *et al.*, 2014). A database, including the RGB and micro-CT images and the phenotypic traits, was set up (Fig. 2H). The definitions and abbreviations of the phenotypic traits are shown in Table 1. The operation of the HCR system is shown in Figs S5 and S6 available at Dryad, and Supplementary Video S1 available at *JXB* online. The image analysis pipeline, core source code, and trait definitions are shown in Fig. S7, Notes S1–S8, and Note S9, respectively, available at Dryad.

**Table 1.** Digital traits extracted by HCR

		Traits	Abbreviation
CT	Tiller traits (raw traits)	Maximum value of total tiller area	MAXTTA
		Mean value of total tiller area	MEANTTA
		Standard deviation of total tiller area	SDTTA
		Maximum value of tiller area/perimeter ratio	MAXTAPR
		Mean value of tiller area/perimeter ratio	MEANTAPR
		Standard deviation value of tiller area/perimeter ratio	SDTAPR
		Convex hull area	CHA
		Total tiller area/convex hull area ratio	THR
		Total tiller area/circumcircle area ratio	TCR
		Total tiller area	TTA
		Tiller number	TN
		Mean value of tiller diameter	MEANTD
		Maximum value of tiller diameter	MAXTD
		Standard deviation of tiller diameter	SDTD
		Mean value of tiller angle	MEANTA
		Maximum value of tiller angle	MAXTA
		Standard deviation value of tiller angle	SDTA
	Tiller growth traits	Absolute growth rate of total tiller area (TTA)	AGRTTAi (i=2,...,9)
		Relative growth rate of total tiller area (TTA)	RGRTTAi (i=2,...,9)
		Absolute growth rate of tiller number (TN)	AGRTNi (i=2,...,9)
		Relative growth rate of tiller number (TN)	RGRTNi (i=2,...,9)
RGB	Plant color trait (raw traits)	Green color value	GCV
	Digital biomass (raw traits)	Green projected area	GPA
		Total projected area	TPA
		Green projected area ratio	GPAR
	Plant architecture traits (raw traits)	Plant compactness	PC1-PC6
		Perimeter/projected area ratio	PAR
		Width of the bounding rectangle	W
		Height of the bounding rectangle	H
		Fractal dimension without image cropping	FDNIC
		Total projected area/bounding rectangle area ratio	TBR
		Height/width ratio	HWR
		Fractal dimension after image cropping	FDIC
		Relative frequencies	F1-F20
	Texture traits (raw traits)	6 histogram texture-related traits	M, SE, S, MU3, U, E
		15 gray-level co-occurrence matrix texture-related traits	T1-T15
	Digital biomass accumulation traits	Absolute growth rate of total projected area (TPA)	AGRTPAi (i=1,...,8)
		Relative growth rate of total projected area (TPA)	RGRTPAi (i=1,...,8)
	Height accumulation traits	Absolute growth rate of plant height (H)	AGRHi (i=1,...,8)
		Relative growth rate of plant height (H)	RGRHi (i=1,...,8)
Manual traits		Shoot dry weight	SDW
		Grain yield	GY

### Growth modeling and yield prediction using phenotypic traits

To test the prediction ability of the different models for total tiller area (TTA) and total projected area of the rice plant (TPA), six models, comprising linear, power, exponential, logarithmic, quadratic, and logistic models, were built and compared. The modeling results were evaluated by comparing the *R*^2^, MAPE, and SD of the absolute percentage error (SD_APE_) values. Statistical analyses of the six TTA and TPA models were developed with LabVIEW 8.6 (National Instruments, Inc., USA). To evaluate the amount of variance in grain yield that can be explained by variation in the early growth traits, linear stepwise regression analysis was performed with the tiller traits, using SPSS software version 13.0 (SPSS Inc., USA).

### Genome-wide association study

A total of 2863169 SNPs with a minor allele frequency ≥0.05 were used for GWAS, and the number of accessions with minor alleles for the SNPs was more than six. Information on these SNPs can be accessed from the RiceVarMap database (http://ricevarmap.ncpgr.cn/v2/). As in previous studies, the genome-wide significance threshold was set at 1.66 × 10^–6^ to control for false positives (Yang *et al.*, 2015). A mixed-model approach with the factored spectrally transformed linear mixed models program FaST-LMM (https://www.microsoft.com/en-us/download/details.aspx?id=52588), running on Linux, was used for the GWAS (Lippert *et al.*, 2011). The kinship coefficient (K) values were defined as the proportion of identical genotypes for the 188165 evenly distributed random SNPs (Xie *et al.*, 2015). Lead SNPs for each trait were determined using the ‘clump’ function of Plink (http://zzz.bwh.harvard.edu/plink/download.shtml), running on Linux (Purcell *et al.*, 2007). Potential candidate genes were obtained using the ‘clump-range’ function of Plink (Purcell *et al.*, 2007). Considering the strong linkage disequilibrium of rice, a region in which the distance of adjacent pairs of associated SNPs was less than 300 kb was defined as the locus (Yang *et al.*, 2015). Haplotypes were determined on the basis of the significant genetic variants.

## Results

### Performance evaluation of tiller traits extraction and measuring efficiency

**Fig. 3.**
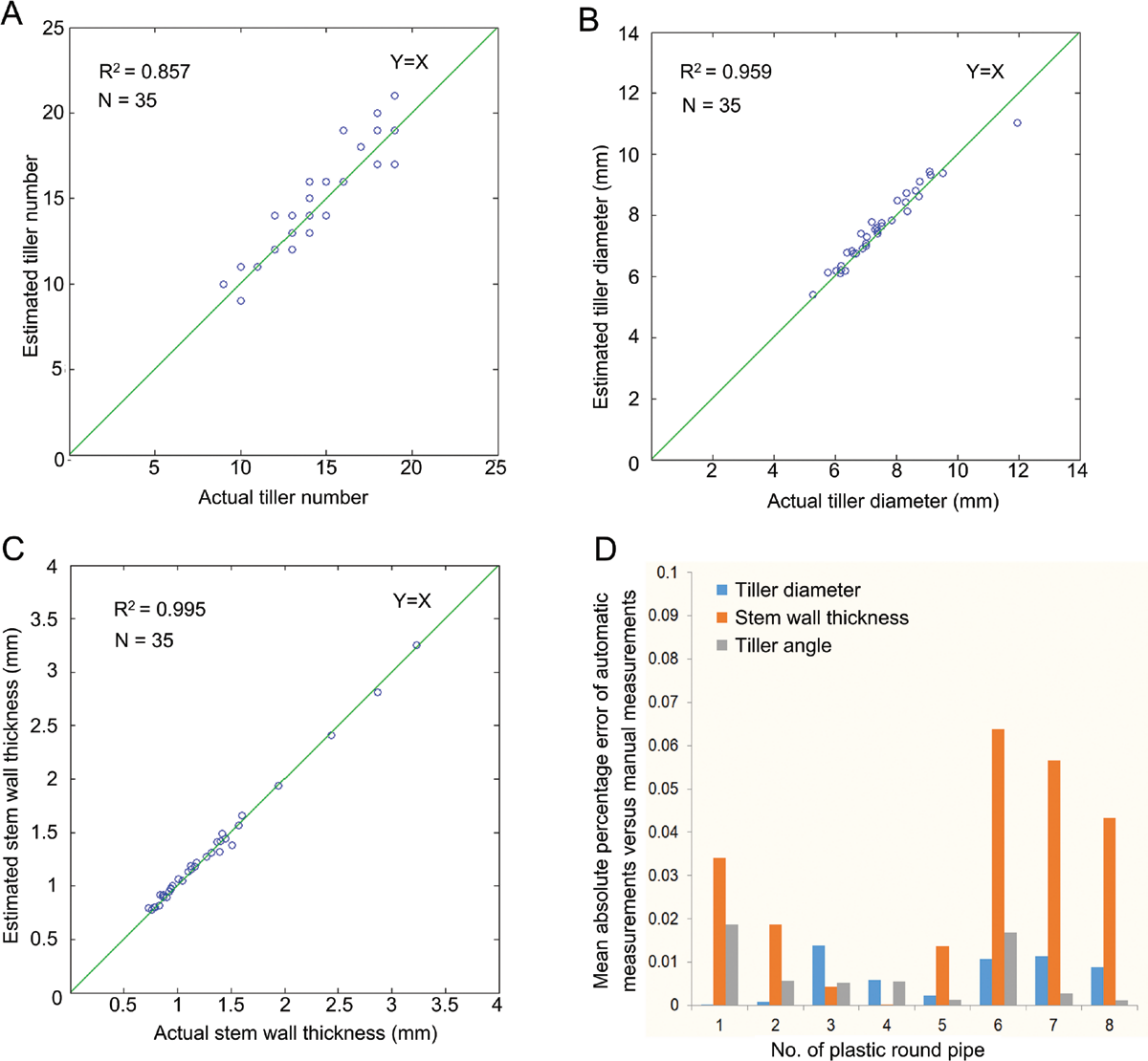
Comparison of automatic digital measurements versus manual measurements. (A–C) Scatter plots of manual measurements versus automatic measurements with the micro-CT unit for (A) tiller number, (B) tiller diameter, and (C) stem wall thickness. (D) Absolute percentage error of automatic measurements versus manual measurements of eight round plastic pipes used to represent rice tillers. To evaluate the accuracy and repeatability of the micro-CT unit, eight round plastic pipes with fixed size were measured manually and digitally 10 times by the micro-CT unit.

Thirty-five plants (Table S4 available at Dryad) were measured both automatically and manually (with manual measurements conducted twice) to verify the accuracy of measurement. The *R*^2^ values of the manual versus automatic measurements were 0.857, 0.959, and 0.995 for tiller number, tiller diameter, and stem wall thickness, respectively (Fig. 3A–C). The MAPE of the manual versus automatic measurements for the same three measurements were 0.78%, 2.96%, and 3.15%, respectively, and the RMSE of the manual versus automatic measurements for the same three measurements were 0.34, 0.30 mm, and 0.05 mm, respectively. To provide an overall picture of the process, the reconstructed images of one rice sample (C055, Sanbaili) at different heights (10.7–54.3 mm distance from the soil surface) are shown in Supplementary Video S2 available at *JXB* online.

In addition, to evaluate the accuracy and repeatability of the micro-CT unit, eight round plastic pipes (fixed in one pot, as shown in Fig. S1 available at Dryad) were measured manually by two people (phenotypic traits are shown in Table S2 available at Dryad) and digitally 10 times by the micro-CT unit (phenotypic traits are shown in Table S3 available at Dryad). The MAPE of the automatic versus manual measurements were approximately 0.02–1.38%, 0–6.38%, and 0.12–1.87% for tiller diameter, stem wall thickness, and tiller angle, respectively (Fig. 3D). The RMSE of the automatic versus manual measurements were approximately 0.07–0.12 mm, 0.02–0.04 mm, and 0.15–1.36°, respectively.

As shown in Fig. S6 available at Dryad, the time taken to acquire one CT image was 0.6 seconds, and 380 CT images were acquired for each plant. Thus, approximately 4 minutes (0.6 seconds×380) were required to complete the CT inspection of each plant. The time taken for one RGB image was 0.6 seconds, and 20 RGB images were acquired at the same time as the CT images. The time taken for manual transfer of each plant was approximately 50 seconds. Therefore, when continuously operated over 24 hours, the total throughput of the HCR system would be 310 plants (~4.6 minutes per plant).

### Screening the dynamic process of rice growth at the tillering and elongation stages

**Fig. 4.**
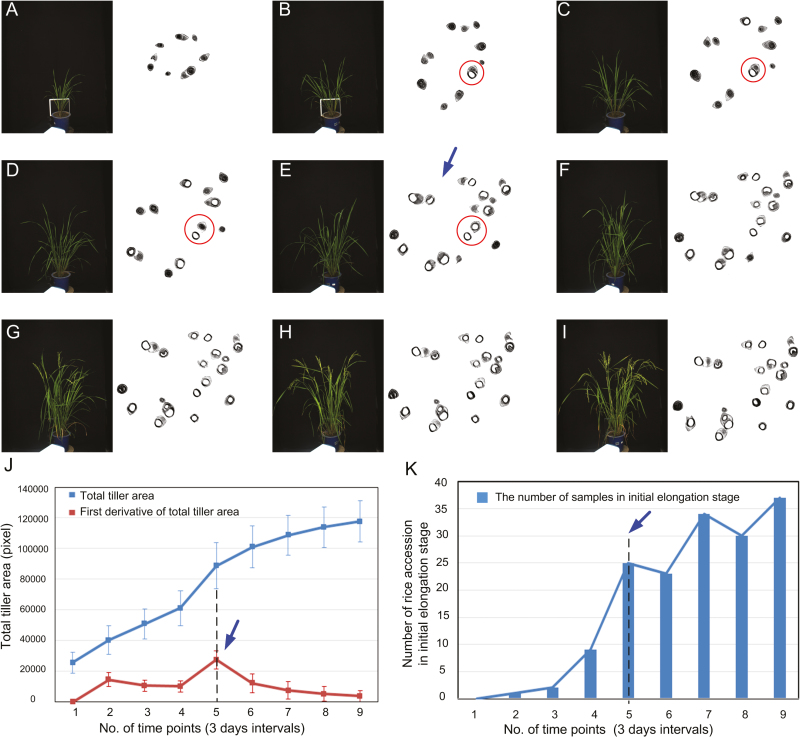
Screening the dynamic process of rice growth at the tillering and elongation stages. (A–I) RGB images and reconstructed CT images at nine sequential growth time points of rice accession (C055, Sanbaili). The red circles indicate dynamic tillering and elongation processes. (J) Changes in total tiller area and the first derivative of total tiller area to reflect the dynamic tiller growth. Error bars represent the SD between the accessions. (K) Distribution of the number of samples (accessions) in the initial elongation stage among the nine time points. The blue arrows indicate the time of most active tiller growth.

During the tillering and elongation stages, 234 plants were automatically measured by HCR at nine time points (every 3 days, from ~41–67 days after sowing). After all phenotypic images and data had been obtained for the nine time points, we screened the dynamic process of rice growth and determined the most active tillering and initial elongation stages. As illustrated in Fig. 4A–I, nine side-view RGB images and nine reconstructed images for each plant were obtained for the following image analysis. The red circle in Fig. 4B–E indicates the dynamic tillering and elongation processes. At time point 2 (Fig. 4B), the first pith cavity appeared, indicating that this plant had progressed into the elongation stage. Subsequently, 10 further elongating tillers could be identified by the point shown in Fig. 4E, with further tillers appearing in subsequent images. The overall (cross-sectional) growth of elongating tillers is quantified as TTA averaged over the whole group of plants (Fig. 4J). The time of most active tiller growth was identified at time 5 (Fig. 4J, blue arrow), where the greatest difference of TTA occurred between successive time points. Tiller growth (quantified as TTA) during the first six periods was relatively faster than that of the later periods. Interestingly, based on the change in the number of rice accessions in the initial elongation stage (Fig. 4K), HCR could detect the variation in the initial elongation stage, which lasted from time point 1 to 9 or even later.

In a previous study, we found that the dark green leaf area ratio was positively correlated with the nitrogen content in leaves (Yang *et al*., 2015). Interestingly, the growth curve of the green color value per unit area (GCV) before time point 5 indicates that the GCV decreased (indicating the presence of more dark green leaves with greater nitrogen content); after time point 5, the GCV increased (indicating more light green leaves with less nitrogen per unit area) (Fig. S8 available at Dryad). The growth curves of 27 representative traits for the tiller and the entire plant are presented in Fig. S8 available at Dryad. The first derivative of plant height (H), plant width (W), and TPA reached the highest value at time point 5, supporting the previous finding that plant vegetative growth reached its highest speed in the active tillering stage (i.e. time point 5). The dynamic growth of one accession (C055, Sanbaili) is shown in Supplementary Video S3 available at *JXB* online.

### Prediction of tiller growth and digital biomass accumulation

**Fig. 5.**
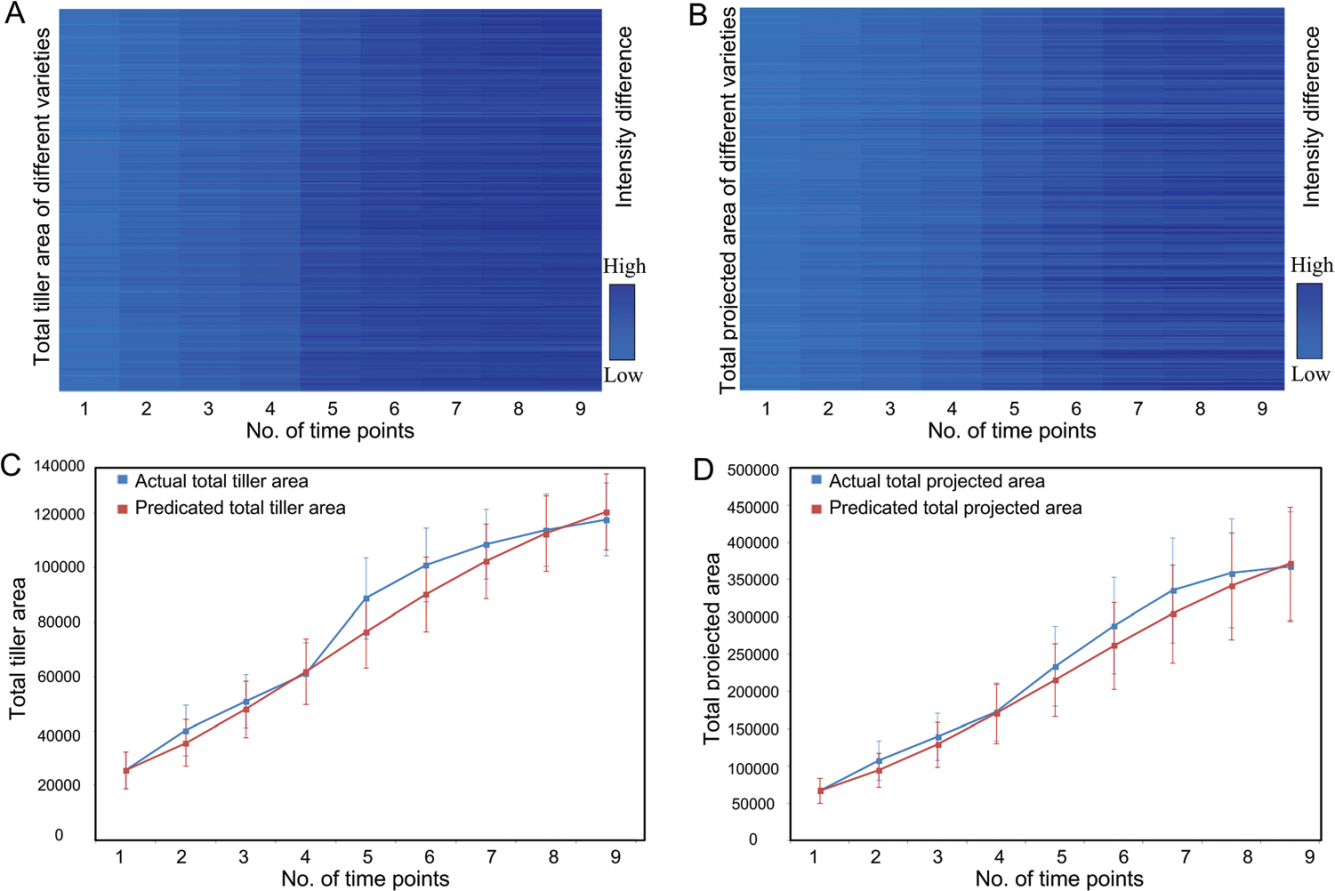
Heatmaps of (A) total tiller area (TTA) and (B) total projected area (TPA) of 234 individual rice plants at nine different time points. (C) Comparison of actual total tiller area (blue line) and predicted total tiller area (red line). (D) Comparison of actual total projected area (blue line) and predicted total projected area (red line). Error bars represent the SE of the TTA or TPA of the 234 samples at each time point.

It would be helpful if a growth model could be designed using the phenotypic data obtained in the early growth stage to predict the final digital biomass. In our previous study, TPA was correlated with actual biomass (Yang *et al.*, 2014). In addition to the manual tiller number count, the TTA extracted by micro-CT can quantify tiller growth more accurately than the tiller number. Fig. 5A and B show the heatmaps of TTA and TPA for the 234 accessions at nine different time points. Here, we tested six models (linear, power, exponential, logarithm, quadratic, and logistic models) of TTA and TPA at the nine points. The results were evaluated by comparing the *R*^2^, MAPE, and SD_APE_ values. As shown in Table S5 available at Dryad, the logistic models of TTA and TPA showed slightly better predictive ability than the other five model types (*R*^2^=0.969 and 0.985, MAPE and SD_APE_ both <6.5%). The actual results versus predicted results of TTA and TPA are shown in Fig. 5C and D, respectively.

### Prediction of grain yield and shoot dry weight from early growth traits

**Fig. 6.**
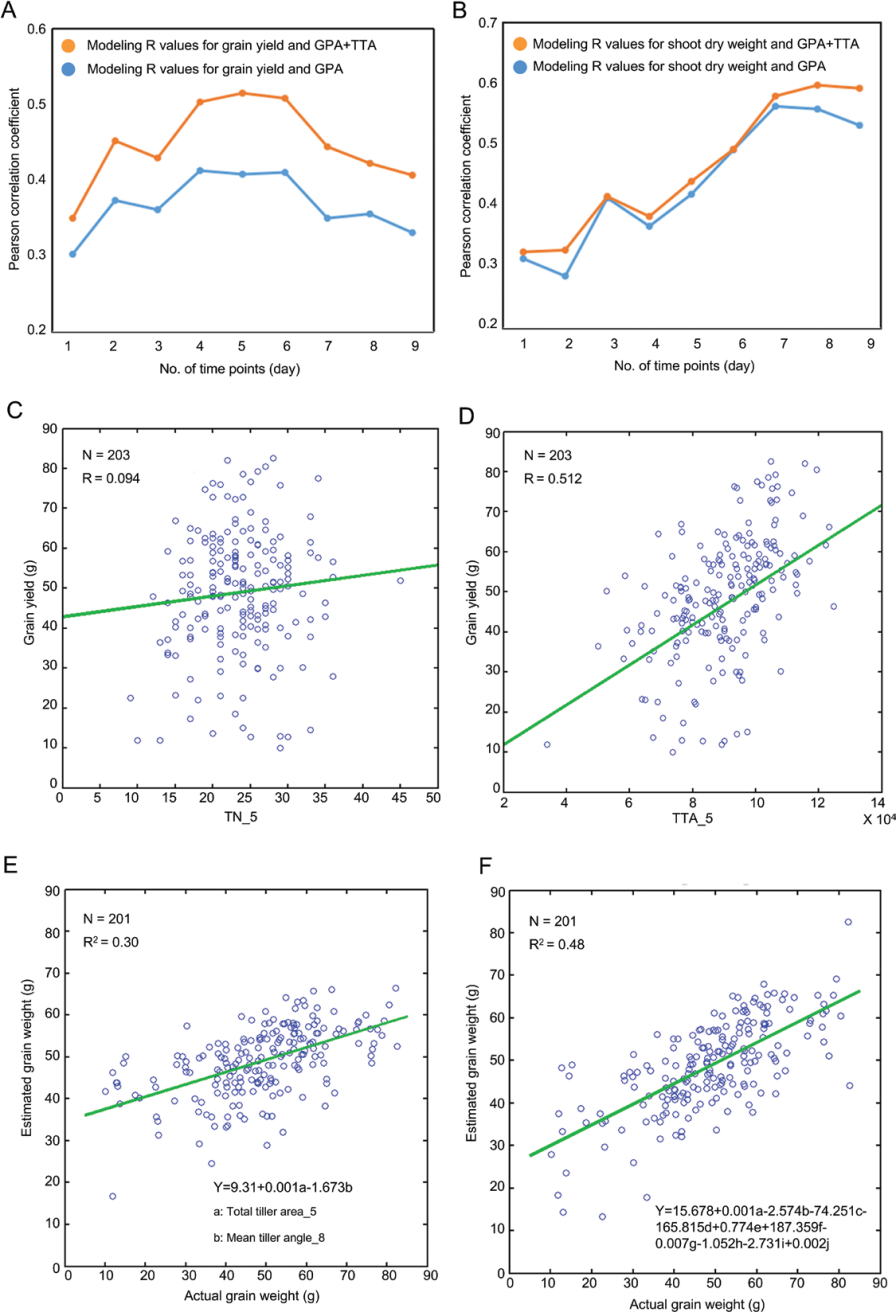
Prediction of grain yield and shoot dry weight. (A) Modeling accuracy change for grain yield at nine time points. (B) Modeling accuracy change for shoot dry weight at nine time points. (C) Scatter plot of tiller number (TN) versus grain yield at the fifth time point. (D) Scatter plot of total tiller area (TTA) versus grain yield at time point 5. (E, F) Scatter plots showing the relationship between the actual and the estimated grain yield using the formula predicted by (E) 2 traits and (F) 10 traits. a, b, c, d, e, f, g, h, i and j represent TTA_5, MEANTA_8, THR_4, FDIC_7, MAXTAPR_7, FDIC_8, SDTTA_5, TN_3, MEANTAPR_2, and MAXTTA_2, respectively (see Table 1 for definitions).

It would benefit rice breeding if the digitally measured phenotypic traits, particularly the traits measured in the early development stages, could be used to predict the final grain yield and shoot dry weight. The *R* value distribution for modeling grain yield in the nine different tillering stages is shown in Fig. 6A. This shows that by adding the TTA, the range of *R* increased from 0.30–0.41 to 0.35–0.51, particularly at time point 5. After time point 5, non-fertile tillers began to grow, providing a possible explanation why the *R* value decreased. Fig. 6B shows that the modeling accuracy for the shoot dry weight was improved by adding a CT trait, TTA. Moreover, we also compared the correlation between TN (tiller number), TTA, and grain yield. The *R* value of TN_5 (TN measured at time point 5) versus grain yield was 0.094 (Fig. 6C), and the *R* value of TTA_5 (TTA measured at time point 5) versus grain yield was 0.512 (Fig. 6D).

When only two phenotypic traits were selected, 30% of the grain yield variance could be explained (Fig. 6E). The two phenotypic traits were both tiller traits, namely TTA_5 and MEANTA_8 [mean value of the tiller angle (MEANTA) measured at time point 8]. We found that the rice yield could be increased by higher TTA_5 and lower MEANTA_8. Up to 48% of the variance in grain yield could be explained by combining 10 traits across all nine time points (Fig. 6F). As shown in Fig. S9 available at Dryad, the *R*^2^ value ranged from 0.34 to 0.46 when combining from three to nine traits.

### Genome-wide association study

**Fig. 7.**
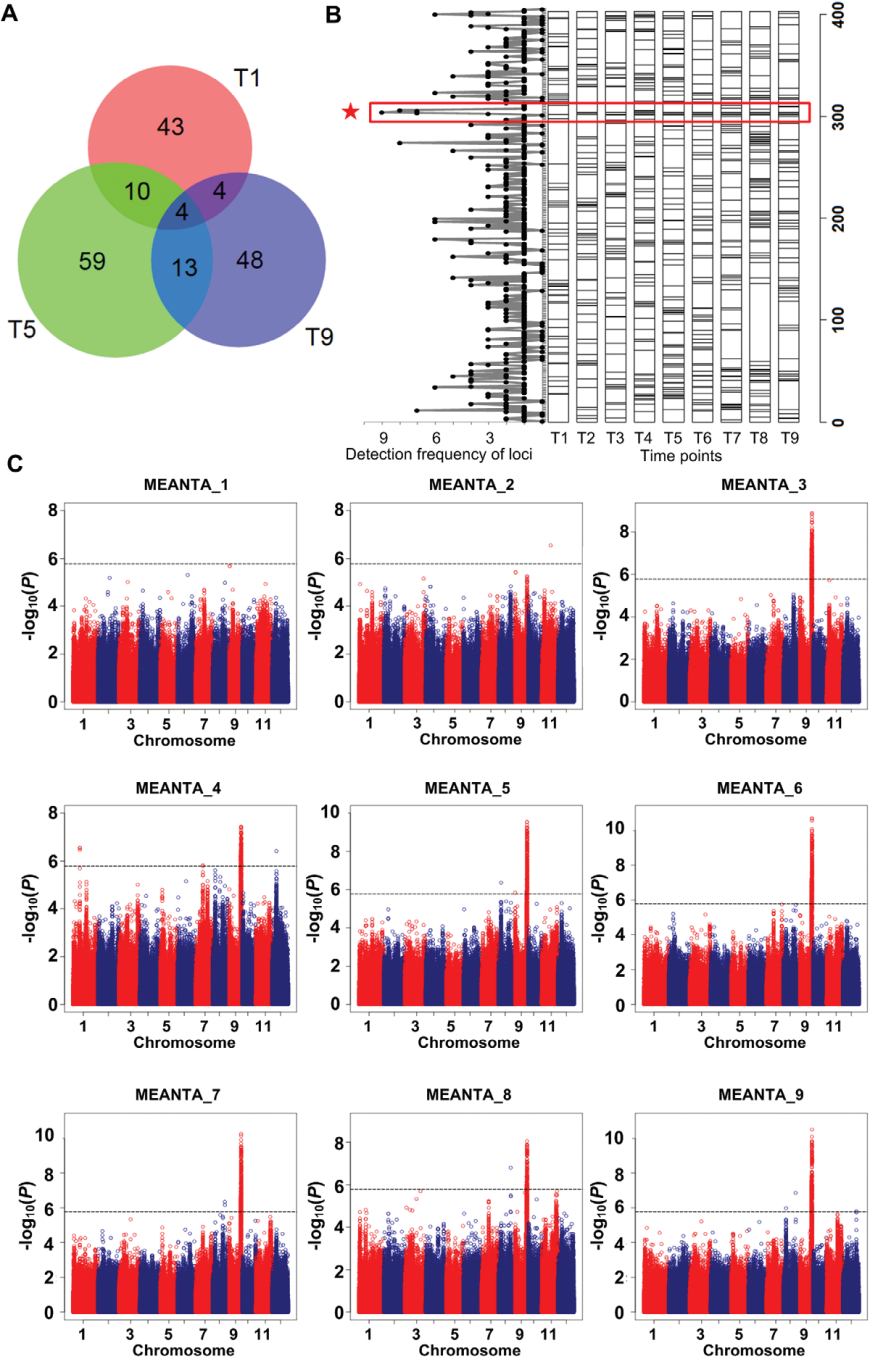
GWAS results of traits at nine time points measured by HCR. (A) Venn diagram showing the number of associated loci at time points 1, 5, and 9. (B) Frequency and distribution of loci associated with traits at the nine time points (T1–T9). (C) GWAS plots of mean of tiller angles (MEANTA) at the nine time points. The strongest association signal on chromosome 9 corresponded to the locus with highest detection frequency (indicated with an asterisk in B).

GWAS of 741 traits (including all 739 traits measured by HCR as well as final grain yield and biomass) identified 402 significantly associated loci (Dataset S2 available at Dryad). In total, 182 and 332 loci were associated with traits measured by micro-CT and RGB, of which 70 and 220 were exclusively detected by micro-CT and RGB, respectively. The numbers of loci associated with traits at different time points ranged from 61 to 87. For example, the numbers of loci at time points 1, 5, and 9 were 61, 86, and 69, respectively; the numbers of overlapping loci at time points 1 and 5, time points 5 and 9, and time points 1 and 9 were 14, 17, and 8; only four loci were detected at all three time points (Fig. 7A). Of the 402 loci, 353 were detected by the raw traits at nine time points and 135 were detected by the derived growth-rate-related traits (Dataset S2 available at Dryad); 86 loci were simultaneously detected by the two groups of HCR traits. Of the 353 loci detected by the raw traits, 191 were detected at only one time point, while the other 162 loci were detected at two or more time points; only one locus on chromosome 9 (locus 302) was detected at all nine time points (Fig. 7B). Furthermore, we found that the chromosome 9 locus was significantly associated with MEANTA measured by micro-CT (Fig. 7C). These results demonstrate the existence of dynamic and static genetic components across rice growth stages.

**Fig. 8.**
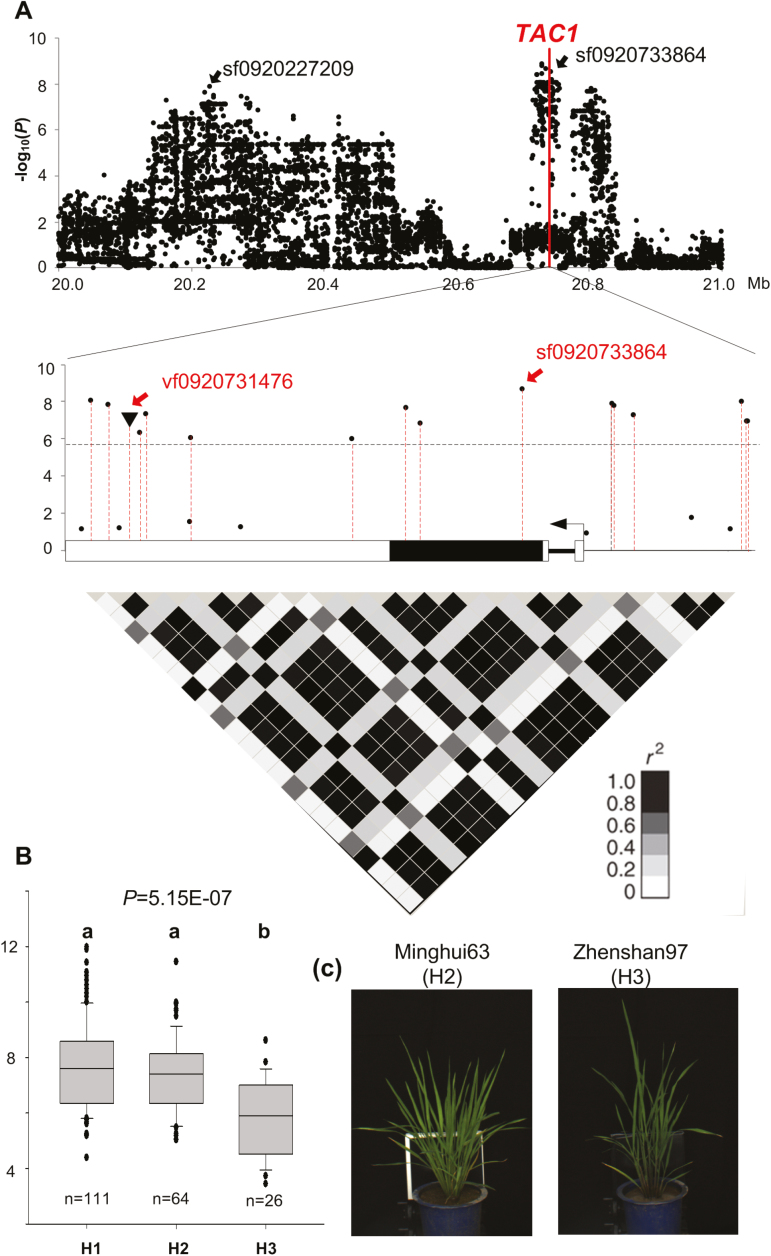
Association analyses of *TAC1* and MEANTA_3. (A) Local Manhattan plots and heatmap showing the level of linkage disequilibrium of the *TAC1* region. (B) Haplotype analyses of *TAC1*. The *P*-value was calculated by ANOVA. Multiple-haplotype comparison was conducted using the least significant difference method; different letters above the boxplots indicate significant differences. (C) Images of two representative varieties, Minghui63 (from the H2 haplotype group) and Zhenshan97 (from the H3 haplotype group).

For locus 302, the linkage disequilibrium decayed slowly (*r*^2^=0.57 between SNPs sf0920227209 and sf0920733864) across a 500 kb region. *TAC1*, a cloned gene known to control tiller angle (Yu *et al.*, 2007), is located at this locus. We found 15 significant SNPs distributed in the 3ʹ-UTR region, coding region, and 1 kb promoter region, and a significant 1 bp indel in the 3ʹ-UTR region (Fig. 8A). All the SNPs within the coding region caused synonymous mutations. Consistent with a previous study (Yu *et al.*, 2007), the variants in the 3ʹ-UTR caused mRNA-level polymorphisms, resulting in tiller angle diversity. Three haplotypes for *TAC1* were found in our association mapping panel, and we found that tiller angles were significantly different among the haplotypes (*P*=5.15 × 10^–7^, ANOVA). Accessions containing haplotype H3 had much smaller MEANTA values (Fig. 8B). Minghui 63 (a known restorer line in hybrid breeding systems) and Zhenshan 97 (a known maintainer line in hybrid breeding systems) contained haplotypes H2 and H3, respectively (Fig. 8C).

**Fig. 9.**
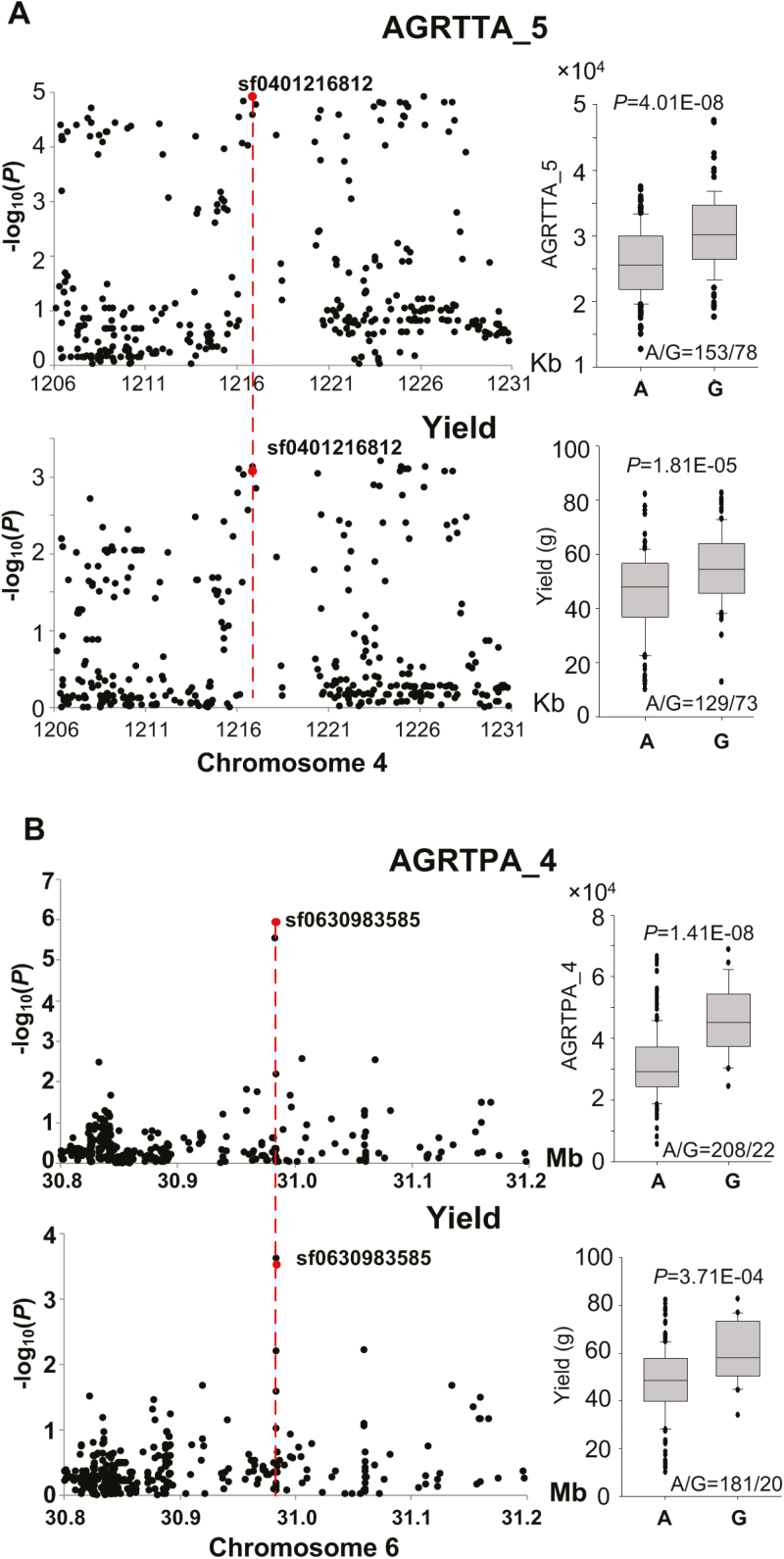
Co-localized loci associated with traits measured by HCR and yield. (A) The locus on chromosome 4 associated with AGRTTA_5 measured by micro-CT (upper panel) and yield (lower panel). (B) The locus on chromosome 6 associated with AGRTPA_4 measured by RGB (upper panel) and yield (lower panel).

Furthermore, we found two loci containing associations with both HCR traits and yield. A lead SNP, sf0401216812, on chromosome 4 was associated with AGRTTA_5 (absolute growth rate of tillering at time point 5) (*P*_MLM_=1.16 × 10^–5^) and yield (*P*_MLM_=8.40 × 10^–4^), and genotype G at the SNP site corresponded to the superior allele for the two traits (Fig. 9A). Another lead SNP, sf0630983585, on chromosome 6 was associated with AGATPA_4 (growth rate of shoot weight at time point 4) (*P*_MLM_=1.14 × 10^–6^) and yield (*P*_MLM_=2.93 × 10^–4^), and genotype G at the SNP site corresponded to the superior allele for the two traits (Fig. 9B). The favorable alleles at the two loci belonged to minor alleles and would be beneficial in breeding for high yield. These results suggest that the vigor of rice plants during the tillering stage could contribute to the final yield.

### Relationship between tiller angle change at the late tillering stage and yield

**Fig. 10.**
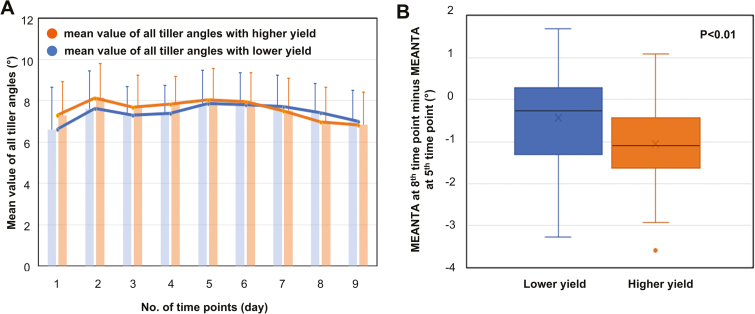
Relationship between tiller angle change at the late tillering stage and yield. (A) Differences in tiller angle change between groups of 50 rice accessions with higher yield and lower yield. Error bars indicate the SD of the mean value of all tiller angles of the 50 accessions per group. (B) Difference of tiller angle change during the late tillering stages (calculated as MEANTA at time point 8 minus MEANTA at time point 5) between the higher-yield and lower-yield groups.

In order to study the relationship between changes in the tiller angle and the final yield, 50 higher-yield accessions (~58–83 g per plant) and 50 lower-yield accessions (~10–41 g per plant) were selected from among the 234 *indica* accessions and analyzed (Fig. 10A). The MEANTA was calculated and is presented as the tiller angle of each accession. First, we compared the MEANTA of the selected 100 accessions across the nine growth stages and found significant differences between time points 1, 5 and 9 (one-way ANOVA, *P*<0.01), which indicated that the tiller angle changed slightly during the elongation stage. There was also a significant change in the tiller angle during the late tillering stages between the higher-yield and lower-yield groups (MEANTA time point 8 minus MEANTA at time point 5), *t*-test, *P*<0.01; Fig. 10B). In addition, from time point 5 to 8, the MEANTA of plants with higher yield fell more than those with lower yield (Fig. 10A). Plants with a larger tiller angle at early elongation and a smaller tiller angle at later elongation will have better light penetration in the canopy later in growth, and this could contribute to their higher yield. The statistical analysis is reported in Tables S6 and S7 available at Dryad.

### Implications of tiller senescence for yield and drought resistance

**Fig. 11.**
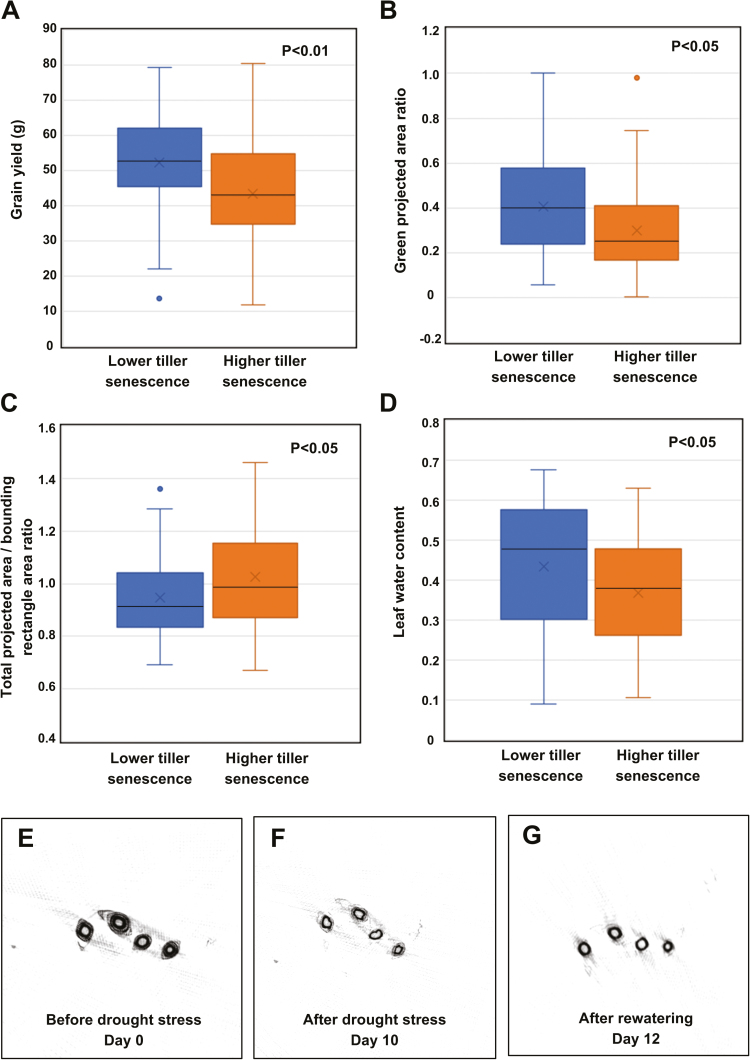
The implications of tiller senescence for yield and drought resistance. Effects of higher and lower tiller senescence on (A) grain yield, (B) green projected area ratio (stay-green trait), (C) total projected area/bounding rectangle area ratio (leaf-rolling trait), and (D) leaf water content. (E–G) Reconstructed tiller images of one rice accession (Zhonghua 11) at three time points: (E) day 0, before drought stress; (F) day 10, after drought stress; (G) day 12, after rewatering.

To investigate whether tiller senescence (defined as in equation 2) is related to yield and drought resistance, we first calculated the tiller senescence of 100 *indica* rice accessions. Then, 50 accessions with higher tiller senescence (~20–42%) and 50 accessions with lower tiller senescence (~0–10%) were compared in terms of their grain yield. Statistical analyses (Table S7 available at Dryad) showed a significant difference for yield between the higher and the lower tiller senescence groups (*t*-test, *P*<0.01). As shown in Fig. 11A, the plants with lower tiller senescence had a slightly higher yield than those with higher tiller senescence.

In our previous study of drought resistance with a large rice population, many drought resistance traits were identified (Guo *et al.*, 2018). In this work, three drought resistance traits, including the stay-green trait (green projected area ratio; GPAR), leaf-rolling trait (total projected area/bounding rectangle area ratio; TBR), and leaf water content were re-analyzed. GPAR_R is calculated as the ratio of GPAR under drought stress to the value before drought stress; a higher GPAR_R indicates higher drought tolerance. TBR_R is calculated as the ratio of TBR under drought stress to the value before drought stress. TBR_R is related to the leaf-rolling trait, and a lower TBR_R indicates higher drought avoidance. The three drought resistance traits were compared in the two tiller senescence groups (i.e. 50 higher and 50 lower-senescence accessions). There were significant differences in the three drought resistance traits between the two tiller senescence groups (*t*-test, *P*<0.05, Table S7 available at Dryad). Interestingly, we found that the rice plants with lower tiller senescence had higher scores for GPAR (implying better drought tolerance), lower TBR (implying better drought avoidance), and higher water content (implying better drought tolerance) (Fig. 11B–D). This finding also indicates that the rice accessions with stable tiller formation will also have better drought resistance, which are both controlled by some common quantitative trait loci (Kim *et al.*, 2017).

In addition, to dynamically screen the changes in tillers under stress, one variety, Zhonghua 11 (*Oryza sativa* L. ssp. *japonica*), was inspected with the HCR system daily for 13 days through progressive drought stress and rewatering. The reconstructed images of tillers during the 13-day period (day 0, before stress; day 1–10, during progressive drought stress; day 11–12, rewatering) are shown in Fig. 11E–G, and Supplementary Video S4 available at *JXB* online. It can clearly be seen that the tillers shrink and wilt after stress, and completely recover after the plant is rewatered. This also indicates that the HCR has the potential to non-destructively quantify tiller changes in large populations of plants under drought stress.

## Discussion

### GWAS of HCR traits reveal the dynamic genetic architecture of tiller-related traits

With the numerous traits extracted by HCR, GWAS detected many significant association signals. The number of loci detected at different time points varied (Fig. 7A). Some loci were identified at a specific time point while others were identified at multiple time points (Fig. 7B), indicating that both dynamic and static genetic components operate during the growth stages of rice. Only one locus on chromosome 9, related to tiller angle, was detected at all nine time points, and the previously identified gene *TAC1* was located at the locus (Fig. 8A). Six significant SNPs and a significant indel were enriched in the *TAC1* 3ʹ-UTR. We found three major haplotypes for the gene in our association mapping panel and observed significant differences in tiller angle among the three haplotypes (Fig. 8B). Although most *indica* accessions in our study harbored the haplotype of the wider tiller angle for *TAC1,* some *indica* accessions harbored the haplotype of the narrow tiller angle, which was not found in previous studies. The polymorphisms in *TAC1* could be further developed for marker-assisted breeding at different densities of planting. Co-localized loci between traits indicate that HCR traits have a higher detection power than does yield, and that the vigor of rice plants during the growth stages contributes to the final yield (Fig. 9A, B).

### Comparison of different phenotyping methods for tiller traits

The traditional methods of determining cereal tiller traits tend to be destructive, labor intensive, and time consuming. Some non-destructive methods of tiller measurement based on two-dimensional (Boyle *et al.*, 2015) or 3D-RGB imaging (Fang *et al.*, 2016) have shown that RGB computer vision approaches can be used to estimate tiller number. However, since the tillers usually overlap each other, RGB imaging is affected by occlusion and cannot accurately detect the innermost tillers even if plants are rotated for multi-angle imaging. The estimation error of RGB imaging methods becomes larger with increasing tiller number because of increasing occlusion. Projected X-ray images obtained by CT overcomes the problem of occlusion, and the tiller numbers can be manually counted (Tracy *et al.*, 2017). However, manual counting is not ideal for measuring large populations, particularly with large tiller numbers. In addition, tiller size and growth traits cannot be measured from projected X-ray images, and micro-CT is needed to measure these traits.

Our approach, using high-throughput imaging of tiller inner structure with good resolution and the corresponding image analysis pipeline for tiller traits, provides the data required for GWAS. In this work, we have integrated CT and RGB imaging within one chamber to develop the HCR system and a corresponding image analysis pipeline to non-destructively extract rice phenotypic traits and provide plant growth data in the vertical and horizontal dimensions (Figs 1 and 2). Compared with other tiller phenotyping methods, HCR has the following advantages:

(i)   The 3D spatial location of tissues can be obtained as traits, such as tiller angle, and can be extracted with more accuracy than by manually measuring them with a protractor, as shown in Fig. 2G. Moreover, the CT system can be easily integrated with an RGB imaging device, allowing additional visual traits (in this study, a total of 75 traits) to be extracted simultaneously (Fig. 2H).
(ii)  The image acquisition time per plant is approximately 4.6 minutes, and the time required for extracting subsequent traits is approximately 2 minutes when combined with graphics processing unit acceleration, thus improving the measurement efficiency per plant.
(iii) Many novel traits, such as TTA, MEANTA, and the absolute growth rate of total tiller area (AGRTTA) can be investigated across time. When comparing the tiller number and grain yield, TTA had a better correlation with grain yield and provided better quantification of tiller growth (Fig. 6C, D).

### Potential application and extension of HCR

The HCR system and the corresponding software were developed to measure the tiller traits of pot-grown rice across its different growth stages, but could easily be extended to many other crops with multiple stems. Small grain cereals, such as barley, wheat, and oats, have a similar canopy structure, and, with some modification, larger grasses such as sugar cane and the biofuel species could be accommodated.

Delivery of the sample to the imaging chamber is done manually, limiting throughput. With conveyor-assisted transport, the total throughput of the HCR system could be increased to 480 plants per day (~3 minutes per plant). Moreover, one could easily envision simultaneous rotation of the X-ray source and panel detector, combined with mounting on an unmanned ground vehicle to acquire similar information from plants in the field.

**Fig. 12.**
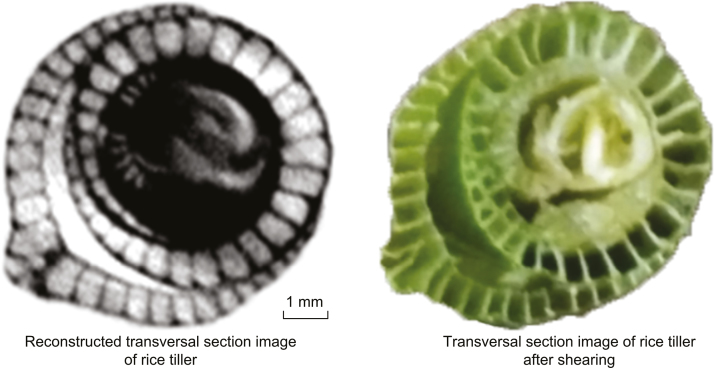
Reconstructed transverse section CT image of a rice tiller at high spatial resolution (30 μm) (left) and transverse section photographic image of the rice tiller after sectioning (right).

Trade-offs between the CT image resolution and scan area currently restrict the application of the system. To provide sufficient FOV for scanning all the tillers in this study, the spatial resolution was set at 97 μm. With better spatial resolution (30 μm; Fig. 12), the tiller and booting development of the main stem could potentially be visualized and their dynamics traced more clearly and non-destructively from the seedling stage to the heading stage; this would be useful to study rice growth and development in the future.

## Conclusions

In this study, we developed an HCR imaging system to extract tiller-related phenotypic traits with high spatial resolution (97 μm) and high efficiency (~310 plants per day). A diverse panel containing 234 *indica* accessions was phenotyped non-destructively at nine time points during the tillering stage, and a total of 739 traits extracted by HCR were used to perform a GWAS. A total of 402 significantly associated loci revealed both dynamic and static genetic components affecting tillering and yield. A major locus associated with tiller angle was detected at nine time points, and the gene *TAC1* was located at the locus. Significant variants associated with tiller angle (evaluated by MEANTA) were enriched in the 3ʹ-UTR of *TAC1.* Three haplotypes for the gene were found, and the tiller angles of accessions containing haplotype H3 were much smaller. Furthermore, two loci contained associations with both HCR traits and yield, which could be beneficial for breeding for high yield and dense planting.

## Supplementary data

Supplementary data are available at *JXB* online.

**Video S1.** Operation of high-throughput micro-CT-RGB imaging system.

**Video S2.** Reconstructed images of one rice sample (C055, Sanbaili) at different heights.

**Video S3.** Dynamic growth of one rice accession (C055, Sanbaili).

**Video S4.** Dynamic change of one rice accession (Zhonghua 11) during drought process (day 0, before stress; days 1–10, after drought stress; days 11–12, rewatering).

## Data availability

All the phenotypic data and images can be viewed and downloaded via the link http://plantphenomics.hzau.edu.cn/checkiflogin_en.action by following these steps: (i) select ‘rice’; (ii) select ‘2015-tiller’ in the year section; (iii) select one of the accession IDs in the ID section and then press ‘search images’; (iv) nine CT images and nine side-view color images can be viewed and downloaded; (v) a similar process can be used to view and download phenotypic traits by pressing ‘search data’. The detailed procedure for the database is shown in Fig. S10 available at Dryad.

## Data deposition

The following tables and figures are available at Dryad Data Repository: doi: 10.5061/dryad.gm18v5f

Data collected from 234 rice accessions, including genotype ID, cultivar name, and all phenotypic traits.

**Dataset S1.** Rice accession information and phenotypic traits (RGB, CT, and manual traits) used in this work.

**Dataset S2.** GWAS results.

**Fig. S1.** Plastic round pipes screened in the HCR system.

**Fig. S2.** Experimental design.

**Fig. S3.** Experimental conditions.

**Fig. S4.** Control flow of image acquisition.

**Fig. S5.** Workflow chart of operation of HCR.

**Fig. S6.** Sequence diagram of the micro-CT-RGB phenotyping system.

**Fig. S7.** Diagram of image processing and feature extraction.

**Fig. S8.** Dynamic growth curve of rice phenotypic traits.

**Fig. S9.** Modeling results of grain yield using different numbers of traits.

**Fig. S10.** Workflow chart of database.

**Note S1.** Source code of sinogram extraction.

**Note S2.** Source code of CT reconstruction.

**Note S3.** Source code of tiller extraction.

**Note S4.** Source code of tiller rotation.

**Note S5.** Source code of tiller diameter extraction.

**Note S6.** Source code of tiller angle extraction.

**Note S7.** Source code of fill holes.

**Note S8.** Source code of tiller area traits extraction.

**Note S9.** Definitions of the traits.

**Table S1.** Main specifications of the micro-CT-RGB inspection unit.

**Table S2.** Manual measurements of eight plastic pipes with two workers.

**Table S3.** Automatic measurements of eight plastic pipes with 10 replications.

**Table S4.** Comparison of automated measurements and manual measurements of rice.

**Table S5.** Comparison of actual TTA/TPA and predicted TTA/TPA with six models.

**Table S6.** Statistical analysis results of one-way ANOVA using SPSS software.

**Table S7.** Statistical analysis results of *t*-test using SPSS software.

## Supplementary Material

Supplementary Video S1

Supplementary Video S2

Supplementary Video S3

Supplementary Video S4
